# Genetic Diversity among Selected *Medicago sativa* Cultivars Using Inter-Retrotransposon-Amplified Polymorphism, Chloroplast DNA Barcodes and Morpho-Agronomic Trait Analyses

**DOI:** 10.3390/plants9080995

**Published:** 2020-08-05

**Authors:** Abdelfattah Badr, Nahla El-Sherif, Sara Aly, Shafik D. Ibrahim, Mohamed Ibrahim

**Affiliations:** 1 Botany and Microbiology Department, Faculty of Science, Helwan University, Cairo 11790, Egypt; abadr@science.helwan.edu.eg; 2 Botany Department, Faculty of Science, Ain Shams University, Cairo 11566, Egypt; elsherif.nahla@sci.asu.edu.eg (N.E.-S.); saraaly@sci.asu.edu.eg (S.A.); 3 Biology Department, Faculty of Science, Taibah University, Madinah 344, Saudi Arabia; 4 Agricultural Genetic Engineering Research Institute (AGERI), Agricultural Research Center (ARC), Giza 12619, Egypt; shafikdarwish2014@gmail.com

**Keywords:** *Medicago sativa* L., genetic diversity, molecular markers, DNA barcoding, IRAP, *mat*K, *trn*H

## Abstract

Alfalfa (*Medicago sativa* L.) is a major forage crop of family Fabaceae and is frequently cultivated in Egypt. The present study is concerned with the genetic discrimination of fifteen alfalfa cultivars from three different countries (Egypt, Australia, and USA) using two molecular approaches: inter-retrotransposon-amplified polymorphism (IRAP) markers and two chloroplast DNA barcodes *mat*K and the *trn*H in addition to the analysis of fifteen morpho-agronomic traits. The genetic relatedness, based on analysis of IRAP marker polymorphism and produced using eleven primers by clustering via principal component analysis (PCA) and multivariate heatmap biostatistical methods differentiated the two Egyptian cultivars EGY1-Ismailia1 and EGY2-Nubaria1 from the three Australian and seven American cultivars, with some distinction of the cv. USA6-SW9720 and cv. AUS4-SuperFast. The results were also supported by the sequence analysis of the *mat*K and the *trn*H genes on the genetic relatedness between eight cultivars. Moreover, it might be suggested that breeding lines from *M. sativa* cultivars may provide novel insights and a better understanding of the domestication of *M. sativa* genetic diversity. The classification of the eight cultivars, as revealed by morpho-agronomic traits, confirmed the close genetic relationship between the two Egyptian cultivars and indicated some resemblance between them and the AUS2-Siri Nafa, whereas the two American cultivars, USA1-Super supreme and USA4-Cuf101, were clearly isolated from a cluster of other three cultivars USA7-SW9628, USA8-Magna901, and USA9-Perfect. The results are useful sources of genetic information for future breeding programs in crop development and open new possibilities of producing *M. sativa* lines harboring high forage quality, productivity, and resistance to biotic and abiotic stresses.

## 1. Introduction

Alfalfa (*Medicago sativa* L.), known as alfalfa and lucerne, is a major important cultivated forage legume crop that was originated in the Caucasus region, and plays a significant role in agricultural sustainability [1]. It is one of the worldwide planted legume crops for forage sources cause of its high biomass yield, highly nutritious preserved forage (viz, pasture, hay, or silage) and wide adaptability to environments. Also, for sustainable cropping systems alfalfa has worthy benefits because of its root formation and perennial life cycle restrict soil erosion. Alfalfa root system forms a symbiosis with *Sinorhizobium meliloti*, the soil bacterium. The latter bacterium species fixes atmospheric nitrogen for increasing soil fertility [2]. Alfalfa has a genome size of 800–1000 Mbp [3] and exists at two polyploids levels; diploid: 2x = 16 and autotetraploid: 4x = 32, where letter “x” is used to define the ploidy level. Alfalfa is cross pollinated and propagated by seeds [1,4]. Like other crops, agronomic and quality traits in alfalfa may be improved using plant materials having rich genetic variation and analysis of morpho-agronomic traits and using marker selected breeding (MSB). Knowledge on the genetic diversity and relatedness between alfalfa cultivars and landraces is limited, but necessary for breeding new genotypes of alfalfa with improved crop quality. In northern Italy, landraces geographically close or of the same commercial ecotype tend to show a greater similarity and landraces are superior to elite varieties of alfalfa in all traits except autumn dormancy and number of florets per inflorescence [5]. Alfalfa germplasm collection was evaluated by multivariate analysis and recorded considerable variation in phenotypic traits [6]. The efficiency of phenotypic and RAPD markers was estimated for diversity assessment of alfalfa accessions from Europe, North America and Australia [7]. In addition to RAPD, other molecular markers have been used to study the genetic diversity of plant genotypes based on DNA polymorphism to differentiate plant species and populations and identify distinct genotypes and superior hybrids from a cross population [8,9,10,11,12,13]. In alfalfa, SSR markers and phenotypic traits revealed similar genetic diversity of 12 feral alfalfa populations originating from southern Manitoba in Canada and ten alfalfa cultivars but both were different from a *Medicago falcata* germplasm [14]. Genetic diversity among 18 non-dormant alfalfa, accessions including ten local ecotypes and eight introduced accessions were studied at the morpho-agronomic and molecular levels using sequence-related amplified polymorphism (SRAP) markers (loci), which are relatively widely distributed across the plant genome [15]. The SRAP data differentiated widely distributed lucerne populations, across different environments and identified good genetic resources for breeding purposes [16]. SRAP was also effectively used to estimate genetic diversity among Tunisian alfalfa genotypes and provided identification and rational use of local and foreign alfalfa populations for breeding programs focusing on the development of new, high-yielding cultivars that are more adapted to the drought conditions in North Africa [17].

Retrotransposons (RTNs) are the most common class of transposable elements in eukaryotes and occur in high copy number in plant genomes [18]. Inter-retrotransposon amplified polymorphism (IRAP) and retrotransposon-microsatellite amplified polymorphism (REMAP) were developed as new retrotransposon-based DNA fingerprinting techniques that produce dominant, multiplex marker systems that examine variation in retrotransposon insertion sites. The IRAPs are good molecular markers due to the high number of copies of retrotransposons in plant genomes and to the fact that they can create new copies [19]. RTNs markers derived from IRAP and REMAP were compared with ISSR, and SSR markers for evaluation of genetic diversity of alfalfa [20]. They concluded that the RTNs markers have the advantage of being easy to assess, their low cost as well as being more informative and polymorphic. The RTNs markers have been used for investigating the genetic diversity in several plants. For example, genomic stability in populations of the young allopolyploid species *Spartina anglica* [21], and the Iranian bread wheat cultivars and breeding lines of wheat [22] and the assessment of genetic diversity and relationships among Triticum species and populations from Iran [23,24]. The genetic diversity of *Bletilla striata* was also assessed by IRAP and the start codon targeted (SCoT) markers [25].

DNA barcoding for plant species identification, based on the use of a standard gene fragment, for plant species identification, was introduced by Hebert et al. [26] and was developed rapidly in the first decade of the 21st century [27]. The plant working group proposed the chloroplast genes *rbc*L and *mat*K as the core barcodes of plant species, as well as the intergenic sequence *trn*H-*psb*A and the nuclear internal transcript spacer sequence (ITS) as supplement barcodes [27]. DNA barcoding may also provide indications of genetic divergence present in populations and assists in comparative investigations of population diversity [28]. In recent years, DNA barcoding have become a useful tool for investigating and monitoring biodiversity and in phylogeny reconstruction and plant evolution studies [29] although alignment of the *trn*H-*psb*A spacer region needs careful attention [30]. Combined universal chloroplast and nuclear DNA sequence targets was applied to barcode the major Mediterranean leguminous crops [31]. The combination of universal chloroplast and nuclear DNA sequence targets was applied to barcode the major Mediterranean leguminous crops [32].

Genetic diversity of *M. sativa* has been frequently characterized using morphological traits [33], sometimes in combination with molecular markers [16]. Warburton and Smith (1993) suggested that there were at least six regional germplasm groups of non-dormant alfalfa among the North African, Arabian, and Indian germplasm [34]. Canonical discrimination functions showed that 75% of total morphological variability among accessions of 18 non-dormant alfalfa was strongly influenced by leaflet shape, stipule shape, and the peduncle, that is, the petiole length ratio [16]. The development of improved alfalfa cultivars may have altered some of the traits of ancestral populations that were associated with inherent yield and could have contributed to the relatively narrow genetic base that is now available for breeding [35,36,37].

The objective of the current study is to use the IRAP markers to differentiate 15 selected cultivars of *M. sativa*. In addition, analysis of fifteen morpho-agronomic traits and DNA barcoding, using the two chloroplast genes *mat*K and *trn*H are used to assess the genetic relatedness and ancestry of the selected alfalfa cultivars. 

## 2. Material and Methods 

### 2.1. Plant Material

Fifteen *M. sativa* cultivars were kindly assessed by ICARDA, research program of crop genetics, currently at the Agricultural Genetic Engineering Research Institute (AGERI), Agricultural Research Center (ARC), Giza, Egypt. The samples include two cultivars from Egypt, three from Australia and ten from the United States of America as listed in Table 1.

### 2.2. Extraction, Purification, and Quantification of Genomic DNA of M. sativa Cultivars

High molecular weight genomic DNA was isolated from 50-100 mg ground and freeze-dried seed meals of M. sativa cultivars based on the procedures of Murray and Thompson (1980) using DNeasy Plant Mini Kit (QIAGEN, Hilden, Germany). The quantity and purity of extracted DNA were assessed spectrophotometrically using the ND-1000 system (Nano-Drop Technologies, Thermo Fisher Scientific Inc., Waltham, MA, USA). DNA manipulation and analysis procedures in terms of quantification and purity analysis were performed according to the Molecular Cloning Laboratory Manual [38]. 

### 2.3. IRAP Primer and IRAP Marker PCR Amplification 

Eleven IRAP primers of those designed by Kalendar and Schulman (2014) were used (Table 2). The primers were synthesized by HVD Vertriebs-Ges. m.b.H. (Vienna, Austria) in 10 nmol stock concentration, delivered in a lyophilized form, rehydrated in sterile water to reach the 100 μM final concentration, and finally stored at −20 °C until used. IRAP-PCR amplification was carried out in 25 μL reaction volume containing 1× PCR master mix buffer supplemented by 1.5 mM MgCl_2_, 25 μM primer, and 75 ng genomic DNA. The amplification process was performed in a Perkin-Elmer/GeneAmp^®^ PCR System 9700 (PE Applied Biosystems, Waltham, MA, USA) programmed to initial denaturation cycle for 3 min at 94 °C followed by 35 cycles, with each cycle consisting of a denaturation at 94 °C for 1 min, annealing at 55 °C for 1 min, and elongation at 72 °C for 1.5 min. The primer extension segment was extended to 7 min at 72 °C in the final cycle. Visualization and documentation of the PCR products were executed by using Bio-Rad ChemiDoc^TM^ MP gel documentation and image system (Cat. no. 1708280).

### 2.4. DNA Barcoding of mat K and trn H Plastid Genes

For the matK and trnH barcoding, the forward and reverse primers sequences given in Table 3 have been used. The PCR amplification of the two gens was performed at initial denaturation of 5 min at 94 °C followed by 40 cycles, each consists denaturation at 94 °C for 30 s, annealing step at 45 °C for 30 s, and elongation at 72 °C for 30 s. The primer extension segment was extended for 7 min at 72 °C in the final cycle. PCR specific products were subsequently electrophoresed on 1.5% *w/v* agarose, stained with EtBr (final concentration 100 µM/L, Sigma-Aldrich^®^, St. Louis, MO, USA) in 1× TBE buffer and visualized as described for the IRAP PCR products. Fractionated PCR amplicons of matK and trnH amplified fragments were recovered from agarose gel using QIAquik^®^ PCR PURIFICATION KIT (Qiagen Inc., Venlo, The Netherlands, Cat. no. 28106) according to the manufacturer’s instructions. Selected and purified amplicons were then cloned into pGEM cloning vector prior to sequencing. 

The DNA sequence was determined by automated DNA sequencing reactions performed using ABI PRISM Big Dye Terminator Cycle Sequencing Ready Reaction Kit (PE Applied Biosystems, Waltham, MA, USA) in conjunction with ABI PRISM (3100 Genetic Analyzer, Macrogene DNA sequencing services, Seoul, Rep. of Korea). DNA sequencing was executed using the Gene Amp 2400 Thermal Cycler. The reaction was performed in 20 µL including 7 µL of terminator ready reaction mix, 0.5 µg of plasmid DNA, and 3.2 pmol M13 universal forward primer. The sequence cycling was programmed to 95 °C for 10 s, 55 °C for 5 s, and 65 °C for 4 min for 30 cycles with thermal ramping according to manufacturer’s recommendations. The sequence of nucleotides was detected automatically by the electrophoresis of the sequencing reaction product on 3100 Genetic Analyzer. The result was provided as fluorimetric scans and revealed nucleotide sequence was assembled using Gblocks software version 0.91b [39,40].

The relationship among the eight M. sativa cultivars, used for phylogenetic relatedness using the DNA barcoding of matK and trnH plastid genes, were also investigated using variation in 15 morpho-agronomic traits as described by Moawed [41]. A list of these traits and their states and description in the eight cultivars are given in Table 5. These traits were regarded as two state characters and scored as a binary matrix and analyzed by the Principle Coordinates Analysis (PCA) to calculate the relatedness of the examined cultivars and determine the impact of the morpho-agronomic traits on the plotting of cultivars in a PCA biplot.

### 2.5. Data Analysis 

The clear distinct reproducible bands generated as IRAP markers were scored as (1) for presence or (0) for absence. The capacity of IRAP primers to discriminate between examined genotypes were analyzed by means of calculating the polymorphic information content (PIC), Resolving power (Rp), Marker Index (MI), and effective multiplex ratio (EMR) values. PIC value for each primer, representing the reflection of the degree of detecting polymorphism, was calculated according to Ghislain et al. [42]. Rp was calculated following the formula of Gilbert et al. [43]. Rp = Σ IB, where IB represents band informativeness which was calculated using: *I*b = 1 − (2 × | 0.5 − p |), where P is the frequency of accessions that harbor bands. EMR and MI values were estimated according to Powell et al. [44]. The IRAP markers matrix were used to calculate the coefficient of genetic similarity matrix and construct a distance tree displaying relationships among cultivars using the unweighted pair group method with arithmetic mean (UPGMA) using PAST, ver. 4.02 software [45]. In addition, principal component analysis (PCA) scatter diagram was constructed based on Dice coefficient genetic similarity matrix by using PAST, ver. 4.02 software [45]. The multivariate analysis was performed through constructing a Heatmap matrix using the module of R software.

Computational analysis of matK and trnH DNA barcoding sequences was carried out. Analysis and assembling of matK/trnH gene sequences for every cultivar was performed via BioEdit software version 7.2.5. Latter sequences were then compared using BLAST function (www.ncbi.nlm.nih.gov/bastn) with all accessible sequences in database. matK/trnH gene sequences were submitted to GenBank (BankIt). Alignment of multiple nucleotide sequences were performed by online ClustalW2 (https://www.ebi.ac.uk/Tools/msa/clustalw2/) and double checked by using MEGAX. Gaps positions were checked and assessed in Gblocks Version 0.91b [39,40]. Phylogenetic analysis was performed in MEGAX software using UPGMA algorithm. Confidence of the clustering was executed in SEQBOOT and followed then by running the data set for 500 replication times in each of the previous programs. The bootstrap values were written on the nodes of generated phylogenetic trees. In addition, PCA biplot based on the morpho-agronomic data matrix was constructed by the multivariate analysis of PAST ver. 4.02 software [45].

## 3. Results

### 3.1. Molecular Characterization and Genetic Relationships as Revealed by IRAP Markers

**Figure 1:**
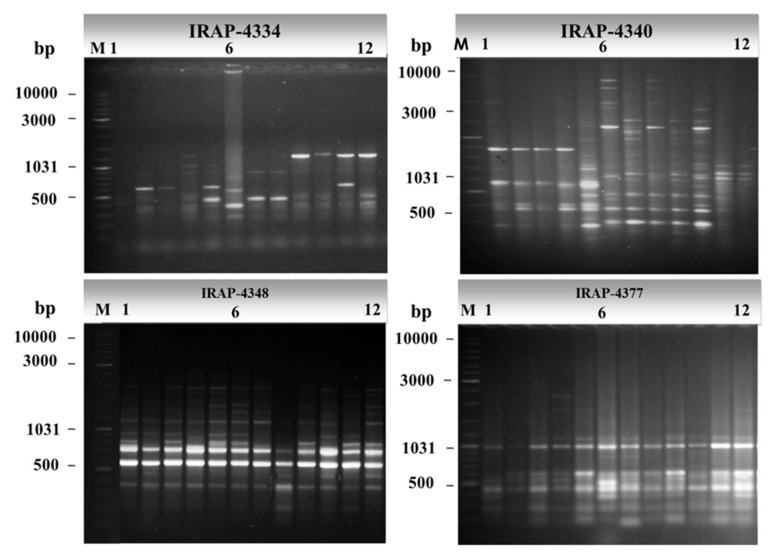
Agarose gel electrophoresis of PCR amplicons of some representative IRAP primers showing polymorphism of IRAP markers. DNA size marker Gene-Ruler 1 Kb plus DNA ladder (lane M) was used as molecular size standards in bps. Numbers from 1 to 12 refer to the sampling numbers of studied cultivars.

The IRAP fingerprinting profiles generated by the eleven primers targeting inter-retrotransposons in 12 alfalfa cultivars are shown in Figure 1. The polymorphism produced by the same 11 IRAP primers is summarized in Table 2. These primers generated a total of 97 amplicons; 85 of these were polymorphic (87.6%). The number of total bands ranged from 3 for primer IRAP-4351 to 21 for primer IRAP-4340; the number of polymorphic bands also varied greatly between 1 and 21 in the cases of IRAP-4351 and IRAP-4340 primers, respectively. Hereby, the average number of polymorphic amplicons was 7.7 per primer. The PIC values varied among the IRAP primers; it ranged from the lowest value of 0.1 for primer IRAP-4351 to the highest value of 0.88 for primer IRAP-4334. Notably, some IRAP primers revealed a pronounced discrimination of 100% polymorphism, including IRAP-4334 and IRAP-4340. IRAP-4334 recorded the highest PIC and EMR values (0.88 and 11, respectively), whereas IRAP-4340 scored the highest RP and MI values (14.2 and 17.36, respectively). On the other hand, primer IRAP-4351 recorded the lowest PIC value (0.1) with the lowest (33%) polymorphism percentage.

**Figure 2:**
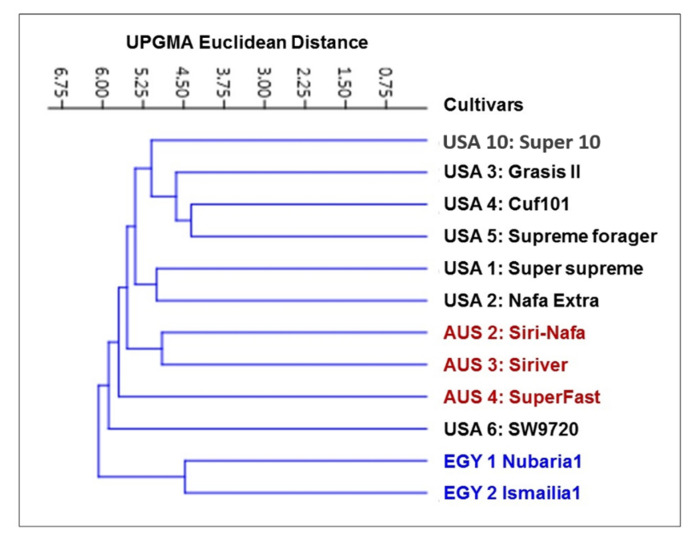
Cluster tree illustrating the relationship of 12 *M. sativa* cultivars based on the analysis of IRAP marker polymorphism, constructed using the Euclidean similarity matrices computed as Dice coefficients and using the unweighted pair group method with arithmetic mean (UPGMA) algorithm in the PAST software.

The IRAP marker data for 12 alfalfa cultivars were used to produce a genetic distance tree based on Dice’s genetic similarity matrix (Figure 2). In this tree, the two Egyptian cultivars, EGY2-Nubaria1 and EGY1- Ismailia1, were clearly separated from the other cultivars, while the USA6-SW9720 and the AUS4-SuperFast cultivars were distinguished as two branches. The other two Australian cultivars were then differentiated from the five USA cultivars; AUS2-Siri Nafa and AUS3-Siriver clustered together and USA10-Super10 was distinct as one branch. USA1-Super-supreme and USA2-Nafa Extra clustered together and the other three cultivars clustered. USA1-Super-supreme and USA2-Nafa Extra clustered together and the other three formed another cluster. The similarity matrices among the 12 *M. sativa* cultivars as computed using Dice’s coefficient based on IRAP molecular marker polymorphism are given in Supplementary Table S1.

**Figure 3:**
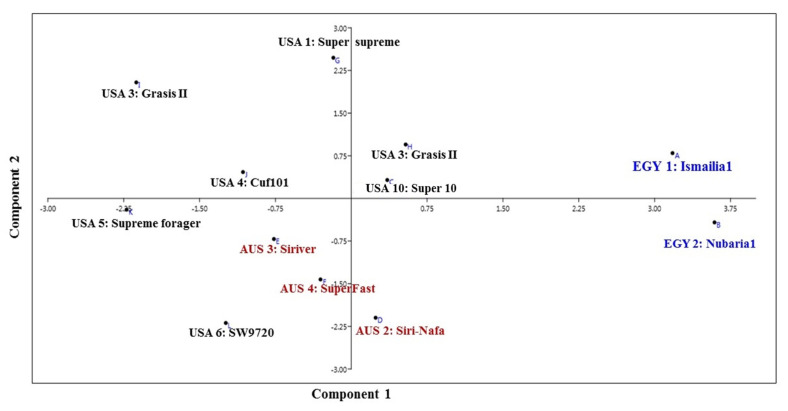
Principal component analysis (PCA) scatter diagram illustrating the genetic diversity expressed by the grouping of 12 *M. sativa* cultivars based on the analysis of IRAP marker polymorphism and by blotting the first two principal components using PAST software.

The PCA analysis reflecting the strength of the IRAP markers to classify the examined cultivars in a PCA scatter plot by plotting of PC1 and PC2, clearly discriminated the two Egyptian cultivars (Figure 3) and revealed the characteristic grouping of the EGY1-Ismailia1 and EGY2-Nubaria1 cultivars. In the same context, the Australian cultivars AUS2-Siri-Nafa, AUS3-Siriver and AUS4-SuperFast were grouped together, but USA10-Super10 was grouped with USA3-Grasis II. On the other hand, the rest of the American cultivars, USA1-Super-supreme, USA4-Cuf101, USA5-Supreme forager and USA6-SW9720, were scattered with some distance to each other.

**Figure 4:**
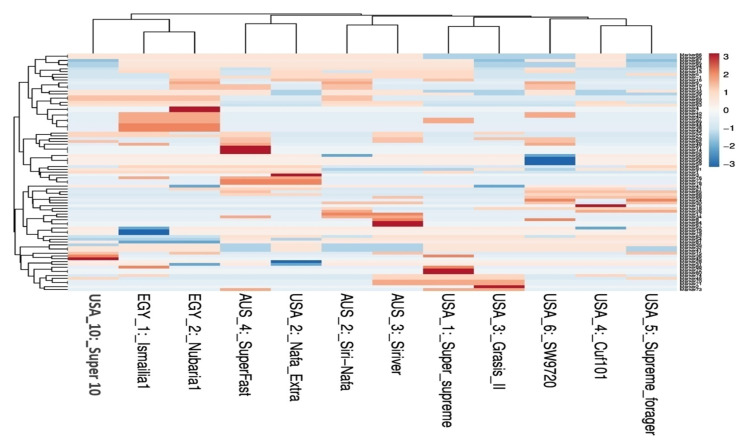
Multivariate heatmap illustrating the genetic diversity of 12 *M. sativa* cultivars based on the IRAP markers constructed using the module of heatmap of R software.

Multivariate heatmap analysis was also used to construct a heatmap using the heatmap module of R software. The twelve cultivars clustered, as shown by the columns, into five clusters of two or three cultivars (Figure 4). The first cluster grouped the Egyptian cultivars together adjacent to USA10-Super10. The cultivars AUS4-SuperFast, USA2-Nafa Extra and the cultivars AUS2-Siri Nafa and AUS3-Siriver are differentiated as two neighboring pairs of cultivars. The other five American cultivars appeared as two neighboring clusters: one of USA1-Super-supreme and USA3-Grasis II and the other of USA4-Cuf101, USA5-Supreme forager and USA6-SW9720.

### 3.2. DNA Barcoding Loci of matK and trnH Sequencing

Two DNA barcoding loci *mat*K and *trn*H from the chloroplast genome were compared for their amplification, sequencing, and successful use in measuring the genetic diversity and phylogeny among eight alfalfa cultivars marked by an asterisk (*) in Table 1. These include the two Egyptian cultivars, AUS2-Siri Nafa, and five USA cultivars (USA1-Super-supreme, USA4-Cuf101, USA7-SW9623, USA8-Magna901 and USA9-Perfect). PCR amplification products of *mat*K and *trn*H regions showed 100% amplification success across all eight cultivars and agarose electrophoresis results demonstrated sharp DNA bands with no byproducts, indicating high specificity of PCR reactions (Supplementary Figure S1).

The produced DNA fragments recorded a size of a 700 bp for both *mat*K and *trn*H. To ensure that the *mat*K and *trn*H sequences, generated in the present study, were of *M. sativa* species, a BLAST function detected that all the sequences strongly matched *Medicago sativa* and *Medicago truncatula mat*K and *trn*H sequences. Pairwise distances were obtained and assessed from the sequences of the conserved *mat*K and *trn*H sequence. Additional information on the genetic variation estimates of DNA barcoding regions of *mat*K and *trn*H loci in eight cultivars of *M. sativa* is given in Table 4, particularly the aligned length, undetermined characters, missing percent, variable sites and the proportion of variable sites, and parsimony informative sites. The evaluation of sequence estimates of DNA barcoding locus of *mat*K locus is given in Supplementary Tables S2 and S3.

**Figure 5:**
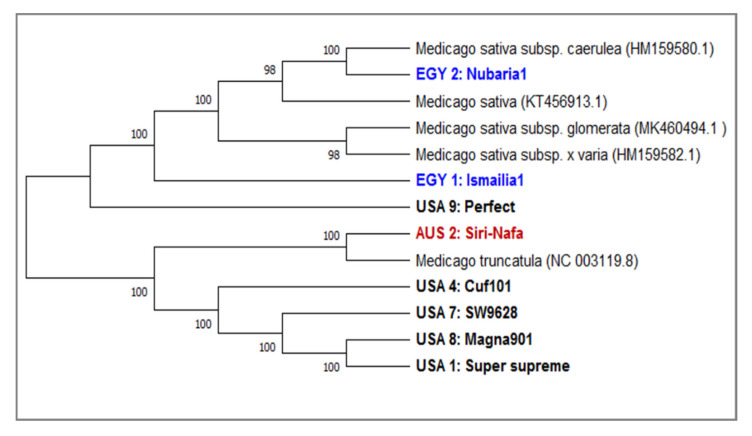
A consensus phylogenetic tree constructed based on the *mat*K DNA barcoding region for nine *M. sativa* cultivars using the MEGAX software. An additional three sequences of *M. sativa* L. *mat*K sequences were used as outgroups. The tree has a branch length of 2.8667 and the bootstrap values shown next to the branches indicate the bootstrap value supporting this node. The evolutionary distances were computed using the Jukes-Cantor method based on base substitutions per site. The ambiguous pairwise deletion option was used.

A phylogenetic tree generated based on *mat*K sequence variation using the UPGMA algorithm clearly discriminated between the eight alfalfa studied sequences (Figure 5). To manifest the accuracy and efficacy of the generated tree, five NCBI-extracted *mat*K sequences belonging to four *M. sativa* (NC_042841.1) and its subspecies x-varia (HM159582.1), glomerate (MK46595494) and caerulea (HM159580.1) as well as *M. truncatula* (NC_003119.1) were used as outgroups. The tree has two major clades: One includes the Egyptian cultivars EGY1-Ismailia1 and EGY2-Nubaria1 along with *M. sativa* NCBI-extracted *mat*K and its subspecies members and USA9-Perfect. The second includes the five USA cultivars, USA4-Cuf101, USA7-SW9623, USA8-Magna901 and USA1-Super-supreme grouped with *M. truncatula*, which is used as an outgroup member. The bootstrap values are very high (98–100%), confirming the validity of the tree branching. A PCA scatter plot based on the of *mat*K barcoding (Supplementary Figure S2) confirmed the classification of the examined cultivars as indicated in the phylogenetic tree generated by the *mat*K locus sequence. This figure revealed the characteristic grouping of EGY1-Ismailia1 and EGY2-Nubaria1 cultivars and the distinction of AUS2-Siri Nafa and USA4-Cuf101. A multivariate heatmap analysis of the *mat*K sequence generated using the heatmap module of R software (Supplementary Figure S3) also confirmed the distinction of the Egyptian cultivars EGY1-Ismailia1 and EGY2-Nubaria1 and AUS2-Siri Nafa from the USA cultivars.

**Figure 6:**
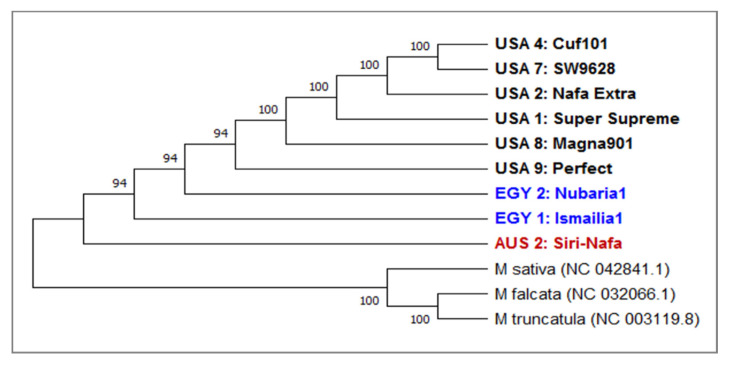
A consensus phylogenetic tree constructed based on *trnH* DNA barcoding regions for nine *M. sativa* cultivars using the MEGAX software. The evolutionary history was inferred using the UPGMA method. The optimal tree has a branch length of 0.905444. The bootstrap values are shown next to the branches. The evolutionary distances were computed using the Jukes-Cantor method and are in the units of the number of base substitutions per site. This analysis involved 12 nucleotide sequences. All ambiguous positions were removed for each sequence pair (pairwise deletion option). There was a total of 60 positions in the final dataset.

On the other hand, similarity matrix revealed a variation between *trn*H-generated sequences among the *M. sativa* cultivars. A phylogenetic tree was constructed based on this variation in eight alfalfa cultivars using *trn*H of *M. sativa* (NC-042841.1; *M. falcata* (NC-032066.1) and *M. truncatula* (NC-003119.1) (Figure 6) as three outgroups.

The tree clearly isolated the three outgroups from the major clade of the eight alfalfa cultivars used for *trn*H barcoding. In this clade, AUS2-Siri Nafa and the two Egyptian cultivars sequences EGY1-Ismailia1 and EGY2-Nubaria1 were clearly isolated from the five American cultivars. A phylogenetic heatmap analysis of the *trn*H sequence generated using the heatmap module of R software (Supplementary Figure S4) also confirmed the distinction of the Egyptian cultivars EGY1-Ismailia1 and EGY2-Nubaria1 and AUS2-Siri Nafa from Australia.

**Figure 7:**
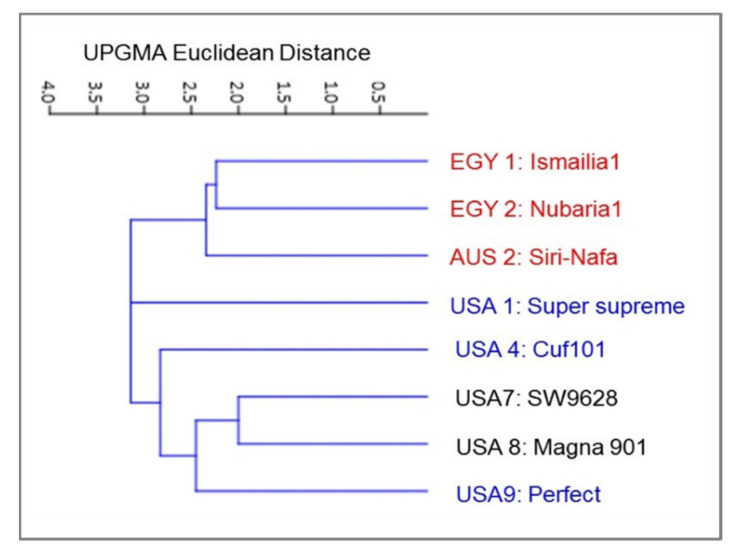
Cluster tree illustrating the genetic distance, based the analysis of 16 morpho-agronomic traits for eight *M. sativa* cultivars using the Euclidean distance and the UPGMA algorithm in the PAST software.

In addition to the genetic diversity among the alfalfa cultivars, the variation in 15 morpho-agronomic traits (Table 5), applicable only to eight *M. sativa* cultivars, was dissected. A distance cluster tree, illustrating the classification of *M. sativa* cultivars, was constructed based on the Euclidean distance of variation in the studied morpho-agronomic traits using the UPGMA algorithm (Figure 7).

**Figure 8:**
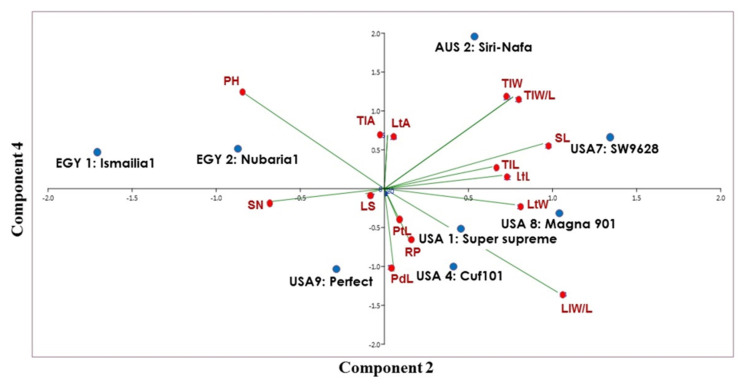
Biplot displays a cluster tree illustrating the genetic distance, based on the analysis of 15 morpho-agronomic traits for eight *M. sativa* cultivars using the Euclidean distance and the UPGMA algorithm in the PAST software.

The clustering of the eight cultivars confirmed the close genetic relationship between the two Egyptian *M. sativa* cultivars. However, AUS2-Siri Nafa was assigned close to these two cultivars, whereas the two cultivars USA1-Super-supreme and USA4-Cuf101 were clearly isolated from a cluster of the other three cultivars, namely, USA7-SW9623, USA8-Magna901 and USA9-Perfect. A PCA biplot, constructed by blotting the PCA 2 and PCA 4, showed the best illustration of the impact of the studied morpho-agronomic traits on the discrimination of the examined cultivars (Figure 8). The affinity of the two Egyptian cultivars and their distinction from the other alfalfa cultivars was most influenced by plant height (PH) and stem number (SN). The distribution of the American cultivars in the biplot was most influenced by the following traits: lateral leaflet width/length (LlW/L), stipule length (SL), lateral leaflet length (mm) (LtL), lateral leaflet width (mm) (LtW), lateral leaflet length (mm) (LtL), and peduncle length (PdL). On the other hand, the distinction of AUS2-Siri Nafa was mostly impacted by the terminal leaflet width (TlW) and terminal leaflet width/length (TlW/L).

## 4. Discussion

In this study, IRAP markers and the chloroplast DNA *mat*K and *trn*H barcodes were used to assess genetic diversity in some M. sativa cultivars currently cultivated in Egypt, USA, and Australia. Genetic diversity has been assessed using 97 markers produced by 11 IRAP primers; with 79.73% polymorphism and an average of 7.72 markers per primer although this number ranges between 3 for IRAP 4351 and 21 for IRAP 4340. Five primers (IRAP 4334, 4370, 4340, 4342, and 3471) produced 100% polymorphism indicating highly variable regions in the genome (Mansour 2008). IRAP fingerprinting also resulted in high polymorphism in different species and reflected intraspecific variations in Mastic tree [46]. The high percentage of polymorphism detected in the present study by IRAP may also indicate a high insertional activity for the assessed RTNs in the genome of *M. sativa* cultivars as reported for wheat [22,23,24,47].

The value of genetic diversity parameters revealed by IRAP markers was used to calculate the genetic diversity of 12 *M. sativa* cultivars using multivariate clustering, PCA, and heatmap analyses indicated that the two Egyptian cultivars and AUS2-Siri-Nafa are distinguished from other cultivars. Close affinity is also clear between the Australian cultivars to each other and the American cultivars to each other with some resemblance between some cultivars as indicated in Figure 2, Figure 3 and Figure 4, in particular between USA10-Super10 and USA3-Grasis II; USA4-Cuf101 and USA5-Supreme forager, as indicated by cluster analysis (Figure 2); and between USA10-Super10 and USA3-Grasis II, as shown in PCA analysis (Figure 3); between USA10-Super10 and EGY1-Ismailia1; and EGY2-Nubaria1 and AUS4-SuperFast and USA2-Nafa Extra (Figure 4). The differentiation of the studied M. sativa cultivars of different countries may be accounted for by former differences in climatic conditions, which might contribute to increasing the genetic variation between cultivars. The findings based on IRAP marker analyses might be attributable to the instability of RTN insertion events as well as to the environmental conditions [48,49].The high level of IRAP markers polymorphism could relate to the outcrossing and the tetraploid nature of alfalfa [20,50,51].

The present study revealed that *mat*K is more variable than *trn*H in sequence variability among the tested *Medicago* cultivars. It has been reported that *mat*K, due to its conservative mode of evolution, is widely used in many phylogenetic studies of flowering plants. It was used to distinguish medicinal plants and validate many herbal products [52,53]. In the current studies, eight alfalfa cultivars were differentiated based on *mat*K sequence variation using five outgroup sequences (Figure 5). The phylogenetic tree generated using five NCBI-extracted *mat*K sequences representing four *M. sativa* subspecies and one of *M. truncatula* supported the outstanding finding of separating the two Egyptian cultivars EGY1-Ismailia1 and EGY2-Nubaria1 along with *M. sativa* NCBI-extracted *mat*K of *M. sativa* and its subspecies members as well as USA9-Perfect which was not included in the IRAP experiment. However, EGY2-Nubaria1 was separated with the *M. sativa* NCBI-extracted *mat*K sequence of the subspecies caerulea, indicating sequence homology between them. In the second clade of four American cultivars, USA4-Cuf101, USA7-SW9623, USA8-Magna901 and USA1-Super supreme grouped with *M. truncatula* and AUS2-Siri Nafa, and the latter two cultivars shared the two most similar *mat*K sequences.

The phylogenetic tree constructed based on the *trn*H sequence showed close homology of the eight examined cultivars as all were clearly isolated from the three outgroups (Figure 6). This may be due to the limited sequence variability in *trn*H sequence in the tested cultivars. In this clade, AUS2-Siri-Nafa and the Egyptian cultivars were clearly isolated from the five American ones in agreement with their clustering based on the morpho-agronomic traits. The phylogenetic heatmap analysis of the *trn*H sequence generated also confirmed the distinction of the Egyptian cultivars EGY1-Ismailia1 and EGY2-Nubaria1 and AUS2-Siri Nafa from Australia.

Despite criticisms of using morphological traits to identify plant cultivars or study their genetic variability, they have proven useful in evaluating genetic diversity in alfalfa [33]. The classification of eight cultivars in the current study confirmed the close genetic relationship between the two Egyptian cultivars and AUS2-Siri-Nafa, whereas the two cultivars USA1-Super supreme and USA4-Cuf101 were clearly isolated from a cluster of other three American cultivars: USA7-SW9623, USA8-Magna901 and USA9-Perfect. Morphological variability among accessions of 18 non-dormant alfalfa was strongly influenced by leaflet shape, stipule shape, the peduncle, and the petiole length ratio [12]. In the current study, the PCA biplot classification of the studied alfalfa cultivars confirmed the affinity between the two Egyptian cultivars, EGY1-Ismailia1 and EGY2-Nubaria1, and their distinction from the other cultivars, but also indicated resemblance to AUS2-Siri Nafa from Australia. The distinction of the two Egyptian cultivars EGY1-Ismailia1 and EGY2-Nubaria1 agrees with the clustering together of local accessions in Saudi Arabia and their differentiation from other origins. Clusters of local accessions at high similarity sometimes correlated with their collection site and morphological traits [16].

Morphological characters are the external expression of the genotype. The plant habit, stem, leaves, flower, fruit and seeds have been used for classification of alfalfa cultivars based on morphological variation [34,54]. Some of the examined cultivars were characterized by morphological traits [41]. The American cultivar USA8-Magna901, originating from DairyLand, America, has a high forage production, a fall dormancy rating of 9, and good growth in winter with a high resistance to nematodes [55]. USA4-Cuf101 is characterized with a high resistance to cold stress, *Fusarium* root rot, and root nodule nematodes. Hereby, USA4-Cuf101 is favored for its high forage production, a dormancy rating of 10, and dry climate resistance. Moreover, USA4-Cuf101 is characterized with a high tolerance to *Fusarium* and *Pythophtora* [55]. USA4-Cuf101 and USA8-Magna901 showed closer affinities to other American cultivars and a distant relationship to the Egyptian and Australian cultivars. Among the Australian cultivars, AUS2-Siri Nafa is characterized by drought tolerance and resistance to root diseases [55].

## 5. Conclusions

The presented results of genetic variation between the studied alfalfa cultivars, based on IRAP, DNA barcoding and morpho-agronomical traits, are considered as potentially useful sources of genetic information in developing future breeding program(s) and opening up new possibilities of producing *M. sativa* lines that have high forage quality, productivity and resistance to biotic and abiotic stress conditions. For these objectives, collection and characterization of genetic resources are required for the development of new cultivars.

## Figures and Tables

**Table 1:** Origin, codes, and names of the alfalfa cultivars as recorded by ICARDA and the Genebank deposited accession numbers for *mat*K and *trn*H genes for eight cultivars of *Medicago sativa*.

No.	Origin	Code ^#^	Cultivar Name	*mat*K Genebank Accession	*trn*H Genebank Accession
1	Egypt (EGY)	EGY1	Ismailia1 * ARC	MN509171	MN509787
2		EGY1	Nubaria1 * ARC	MN509788	MN610248
3	Australia (AUS)	AUS2	Siri-Nafa * Australia	MN509173	MN610246
4		AUS3	Siriver Australia	NA	NA
5		AUS4	SuperFast Saudi Arabia	NA	NA
6	United States of America (USA)	USA1	Super-supreme * California	MN509175	MN509790
7		USA2	Nafa Extra USA	NA	NA
8		USA3	Grasis II California	NA	NA
9		USA4	Cuf101 * California	MN509177	MN610247
10		USA5	Supreme forager California	NA	NA
11		USA6	SW9720 California	NA	NA
12		USA7 ^×^	SW9628 *- California	MN610249	MN509792
13		USA8 ^×^	Magna901 * Diary Land	MN509174	MN509789
14		USA9 ^×^	Perfect * California	MN509172	MN610245
15		USA10	Super10 California	NA	NA

^#^ Three digit codes used in this study are according to official ISO country codes that were listed on http://www.nationsonline.org/oneworld/country_code_list.htm and http://www.worldatlas.com/aatlas/ctycodes.htm. * Refers to the cultivars used for DNA barcoding. ^×^ Refer to cultivars not used for inter-retrotransposon-amplified polymorphism (IRAP) markers.

**Table 2:** IRAP primer names, sequences, total number of amplicons (TNAs), monomorphic amplicons (MAs), polymorphic amplicons (PAs), percentage of polymorphism (%P), polymorphism information content (PIC), resolving power (RP), effective multiplex ratio (EMR), and marker index (MI) as revealed by IRAP profiles in 11 *M. sativa* L. cultivars.

No.	Primer Name	Sequence (5′–3′)	TNAs	MAs	PAs	% P	PIC	RP	EMR	MI
**1**	IRAP4352	ACCCGGAAGGGCGGTTCATGCAA	9	1	8	89	0.78	6.5	7.11	5.57
**2**	IRAP 4334	CCATGGCGAGCAGATGTGCT	11	0	11	100	0.88	7	11	9.67
**3**	IRAP 4348	TTAGATGAAACCAACGATCCCAAGGCT	13	3	10	77	0.52	16	7.69	4.02
**4**	IRAP 4370	ATGCCGTATTCTCAGCATCC	14	0	14	100	0.81	10.2	14	11.41
**5**	IRAP 4377	CGTACCCTTTTAAGGGATCAAAACC	9	2	7	78	0.53	10.8	5.44	2.91
**6**	IRAP 4351	CAGGCAAGAATGAGCGTCTC	3	2	1	33	0.1	5.7	0.33	0.03
**7**	IRAP 4340	ATGGTTGTCGAAACTCCAGC	21	0	21	100	0.83	14.2	21	17.36
**8**	IRAP 4342	GATTGCAAAGCCTATTTCGCTG	4	0	4	100	0.59	4.3	4	2.34
**9**	IRAP 4375	ATCGCTCCGGGTGCCTAACAC	4	2	2	50	0.28	6.7	1	0.28
**10**	IRAP 3471	ATCGCTCCGGGTGCCTAACAC	5	0	5	100	0.61	5.5	5	3.06
**11**	IRAP 4357	TGACATTTGTGGCACTTTCTGGCGT	4	2	2	50	0.28	6.7	1	0.28
Total			97	12	85	NA	NA	NA	NA	NA
Mean					7.72	79.73	0.56	8.50	7.05	5.17

Underlined values denote the minimum and maximum value of each estimated parameter.

**Table 3:** Primer codes and sequences for barcoding the *mat*K and *trn*H genes.

Primer Code	Sequence	Product Size	Reference
*trn*H-F	5′-GTTATGCATGAACGTAATGCTC-3′	700 bp	[31]
*trn*H-R	5′-CGCGCATGGTGGATTCACAATCC-3′		[32]
*matK*-472F	5′-CCCRTYCATCTGGAAATCTTGGTTC-3′	700 bp	[33]
*matK*-1248R	5′-GCTRTRATAATGAGAAAGATTTCTGC-3′		

**Table 4:** Genetic variation estimates of DNA barcoding of the *mat*K and *trn*H genes in the eight cultivars of *M. sativa*.

Gene Name	Aligned Length	Undetermined Characters	Missing Percent	Variable Sites	Proportion Variable Sites	Parsimony Informative Sites	Proportion Parsimony Informative	AT%	GC%
*mat*k	911	863	13.53	327	0.359	194	0.213	0.66	0.34
*trn*H.	755	521	8.63	31	0.041	4	0.005	0.64	0.36

**Table 5:** List of 16 morpho-agronomic traits and their state in the eight alfalfa cultivars used for phylogeny of these cultivars using *mat*K and *trn*H gene sequences.

Characters and Their Abbreviations	EGY1: Ismailia1	EGY2: Nubaria1	AUS2: Siri-Nafa	USA1: Super Supreme	USA4: Cuf101	USA7: SW9628	USA 8: Magna 901	USA9: Perfect
Resistance to pests: RP	sensitive	sensitive	resistant	sensitive	sensitive	sensitive	sensitive	sensitive
Plant height (cm): PH	≥70	≥70	<70	≥70	≥70	<70	<70	<70
Stem number: SN	<4	<4	<4	≥4	≥4	<4	<4	<4
Petiole length (mm): PtL	<20	<20	<20	≥20	<20	<20	<20	<20
Petiole hairiness: PtH	glabrous	hairy	hairy	glabrous	glabrous	glabrous	hairy	glabrous
Terminal leaflet length (mm): TlL	<20	≥4	<20	≥4	<20	≥4	<20	<20
Terminal leaflet width (mm): TlW	<8	≥8	<8	≥8	<8	≥8	<8	<8
Terminal leaflet width/length: TlW/L	<0.4	≥0.4	<0.4	≥0.4	<0.4	≥0.4	<0.4	<0.4
Lateral leaflet length (mm) LtL	≥18	≥18	≥18	<18	<18	≥18	≥18	≥18
Lateral leaflet width (mm): LtW	≥5	≥5	≥5	≥5	<5	≥5	≥5	≥5
Lateral leaflet width/length: LlW/L	<0.32	<0.32	≥0.32	≥0.32	<0.32	≥0.32	≥0.32	<0.32
Terminal leaflet apex shape: TlA	mucronate	mucronate	mucronate	mucronate	mucronate	mucronate	mucronate	hooked
Leaf stipule shape: LS	hairy	leafy	leafy	leafy	leafy	leafy	hairy	leafy
Stipule length (mm): SL	≥9	<9	<9	<9	<9	<9	≥9	<9
Peduncle length (mm): PdL	<18	≥18	<18	<18	<18	≥18	≥18	≥18

## Supplementary Materials

The following are available online at https://www.mdpi.com/2223-7747/9/8/995/s1: Figure S1: Agarose gel electrophoresis of PCR products showing amplification of DNA loci of *mat*K and *trn*H genes in eight *M. sativa* cultivars; Figure S2: PCA scatter diagram illustrating genetic diversity based on the analysis of *mat*K DNA barcoding regions for nine *M. sativa* cultivars by blotting the first two principal components using PAST software; Figure S3: Multivariate heatmap illustrating the genetic diversity of nine *M. sativa* cultivars (denoted by asterisks in Table 1) and four outgroups, using the module of heatmap of R software based on the *mat*K barcoding sequence and five NCBI-extracted *mat*K sequences belonging *M. sativa* and its subspecies x-varia, glomerate and caerulea, as well as *M. truncatula;* Figure S4: Multivariate heatmap illustrating the genetic diversity of nine *M. sativa* cultivars (denoted by asterisks in Table 1) and four outgroups, using the module of heatmap of R software based on trnH DNA barcoding regions and three NCBI-extracted trnH sequences belonging to *M. sativa* and its subspecies glomerate, and caerulea; Table S1: Similarity matrix among 12 *M. sativa* cultivars, as computed using Dice’s coefficient based on IRAP molecular marker polymorphism; Table S2: Evaluation of *mat*K DNA barcoding region; Table S3: Evaluation of *trn*H DNA barcoding region.
