# Supplementary material for: Genetic Diversity among Selected Medicago sativa Cultivars Using Inter-Retrotransposon-Amplified Polymorphism, Chloroplast DNA Barcodes and Morpho-Agronomic Trait Analyses
Source: Plants (Basel). 2020 Aug 5;9(8):995. doi: 10.3390/plants9080995 (PMC7464242; doi:10.3390/plants9080995)
Supplement: Supplementary file 1 [file plants-09-00995-s001.pdf]

Supplementary materials

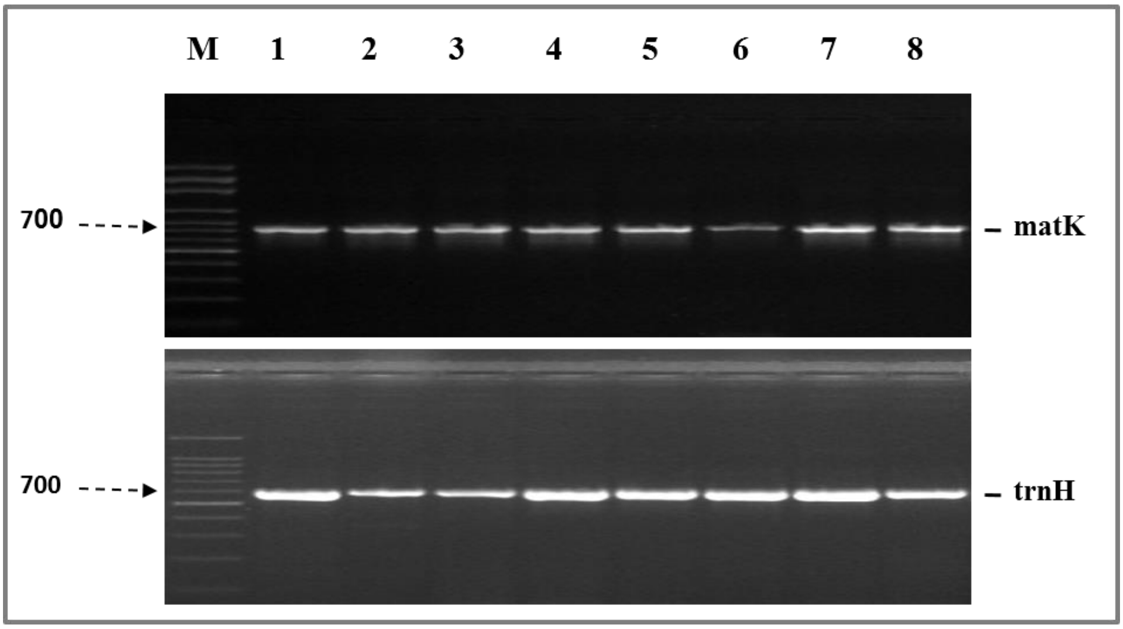

Supplementary Figure 1. Agarose gel electrophoresis of PCR products showing amplification of DNA loci of *mat*K and *trn*H genes in eight *M. sativa* cultivars. Black arrows indicate the molecular size of amplified *matK* and *trn*H loci. The cultivars denoted by an asterisk (*) in Table 1.

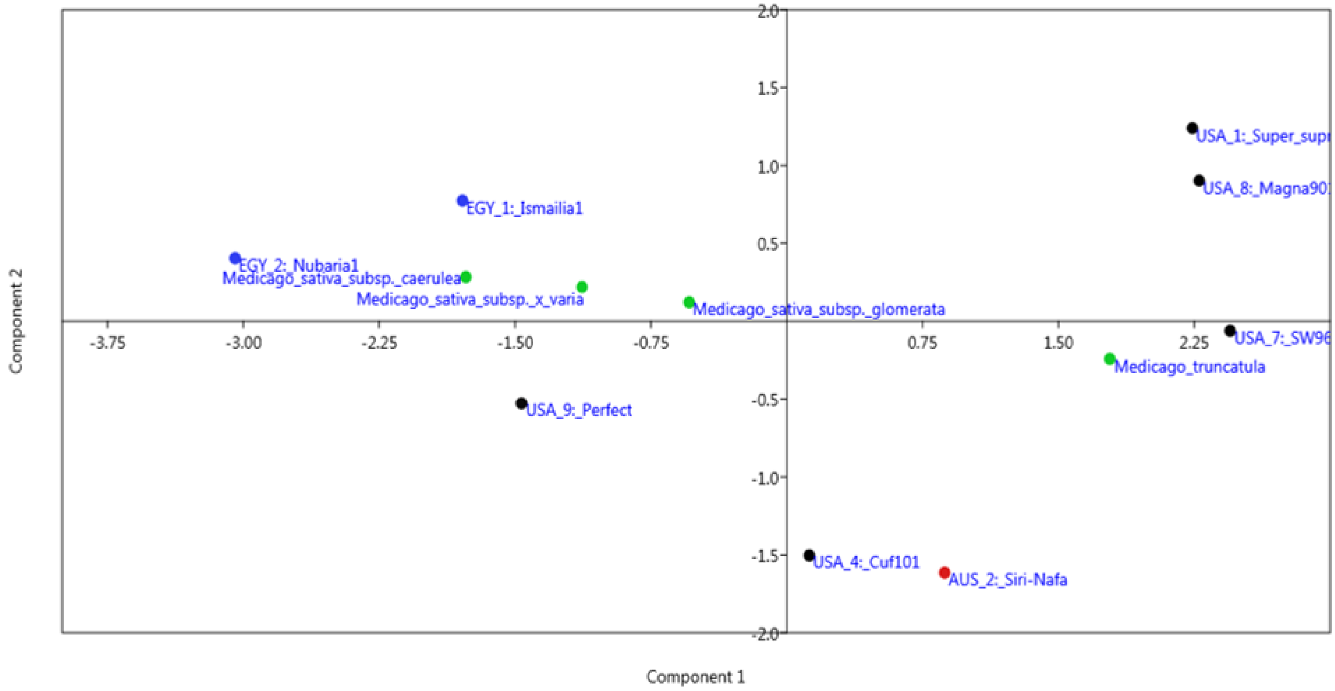

Supplementary Figure 2. PCA scatter diagram illustrating the genetic diversity, based on the analysis of *mat*K DNA barcoding region for nine *M. sativa* cultivars by blotting the first two principal components using PAST software.

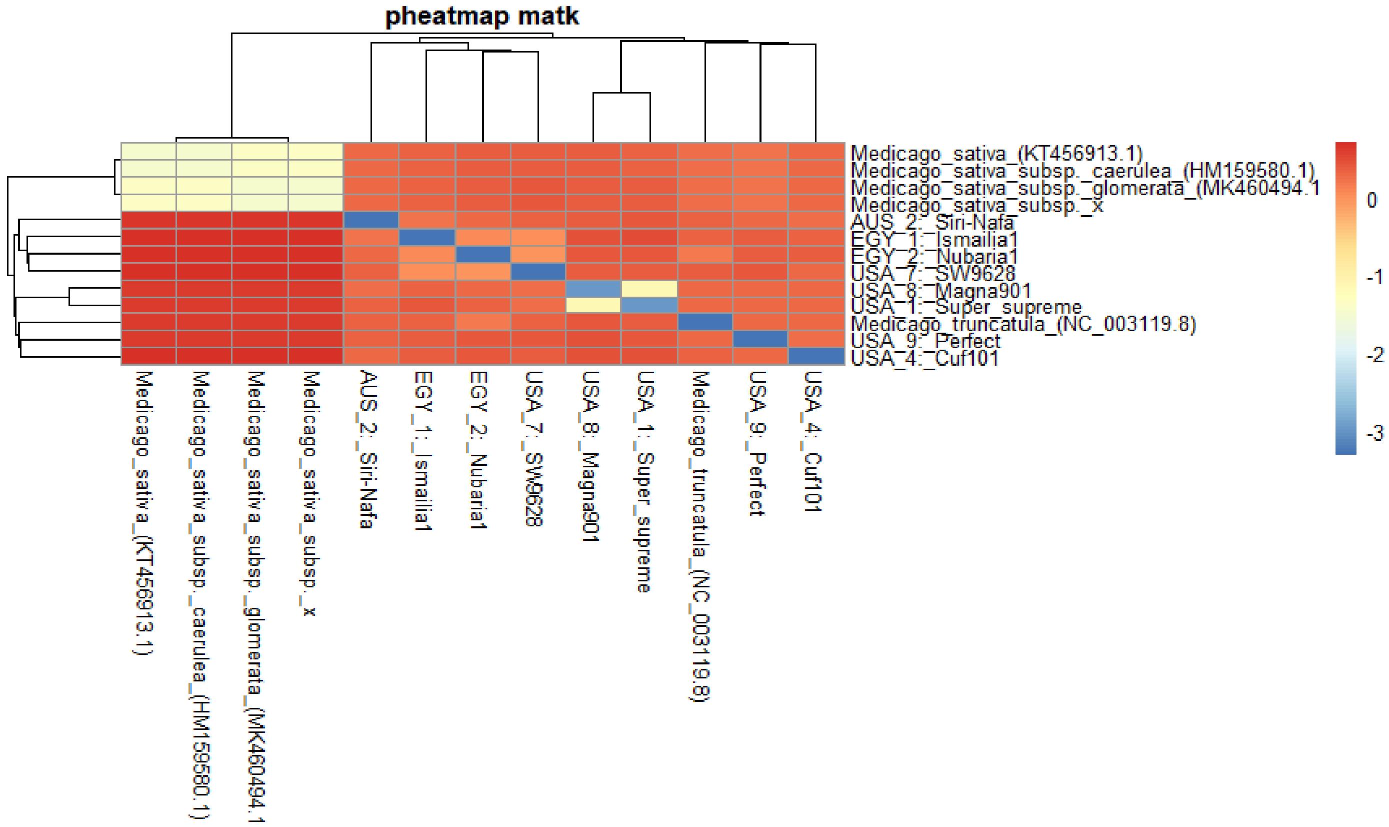

Supplementary Figure 3. Multivariate heatmap illustrating the genetic diversity of nine *M. sativa* cultivars (denoted by asterisk Table 1) and four outgroups, using the module of pHeatmap of R software based on the *mat*K barcoding sequence and five NCBI-extracted *mat*K sequences belonging *M. sativa* and its subspecies x-varia; glomerate and caerulea and *M. truncatula.*

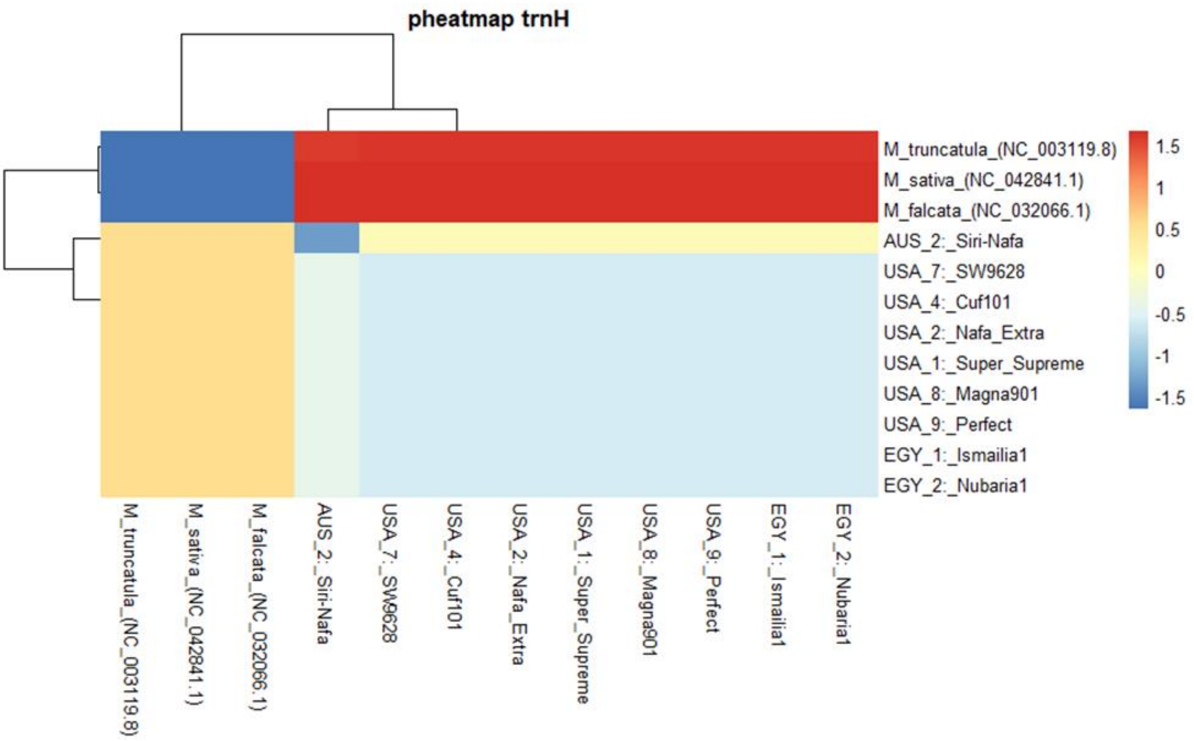

Supplementary Figure 4. Multivariate heatmap illustrating the genetic diversity of nine *M. sativa* cultivars (denoted by asterisk Table 1) and four outgroups, using the module of pHeatmap of R software based on *trnH* DNA barcoding region and three NCBI-extracted *trnH* sequences belonging to *M. sativa* and its subspecies glomerate; and subspecies caerulea.

Supplementary Table 1. Similarity matrix among 12 *M. sativa* cultivars, as computed using Dice coefficient based on IRAP molecular markers polymorphism.

***M. sativa* cultivars**	**EGY 1: Ismailia1**	**EGY 2: Nubaria1**	**AUS 1: Super10**	**AUS 2: Siri-Nafa**	**USA 1: Super supreme**	**AUS 3: Siriver**	**AUS 4: SuperFast**	**USA2: Nafa Extra**	**USA 3: Grasis II**	**USA 4: Cuf101**	**USA 5: Supreme forager**	**USA 6: SW9720**
EGY1: Ismailia1	1											
EGY2: Nubaria1	0.244	1										
AUS1: Super10	0.298	0.341	1									
AUS2: Siri-Nafa	0.448	0.37	0.341	1								
USA1: Super supreme	0.567	0.586	0.385	0.355	1							
AUS3: Siriver	0.522	0.586	0.416	0.48	0.4	1						
AUS4: SuperFast	0.4	0.48	0.416	0.416	0.341	0.532	1					
USA2: Nafa Extra	0.48	0.4	0.432	0.492	0.48	0.326	0.326	1				
USA3: Grasis II	0.497	0.623	0.298	0.48	0.4	0.464	0.284	0.448	1			
USA4: Cuf101	0.464	0.549	0.355	0.416	0.4	0.37	0.4	0.298	0.312	1		
USA5: Supreme forager	0.567	0.549	0.298	0.355	0.4	0.432	0.464	0.416	0.257	0.257	1	
USA6: SW9720	0.549	0.497	0.497	0.4	0.549	0.48	0.549	0.532	0.416	0.416	0.326	1

Supplementary Table 2. Evaluation of *mat*K DNA barcoding region.

qseqid	sseqid	pident	length	mismatch	gapopen	qstart	qend	sstart	send	evalue	bitscore
Nubaria1	YP_009141593.1	100	288	0	0	1	864	144	431	0	559
Magna901	YP_009662972.1	100	278	0	0	1	834	154	431	0	570
Super_Supreme	YP_009662972.1	100	266	0	0	1	798	154	419	0	548
Sirinafa	CED95595.1	80.46	87	17	0	2	262	153	239	2.34E-53	145
Cuf101	ALH22171.1	82.52	143	21	2	27	455	25	163	3.34E-71	223
Perfect	QBJ26779.1	87.62	105	13	0	88	402	36	140	9.01E-56	187
SW9623	BAO66622.1	100	229	0	0	1	687	39	267	1.63E-165	467
Ismailia1	CCI55129.1	100	288	0	0	1	864	147	434	0	587

Sequence estimates of DNA barcoding locus of *mat*K gene were analyzed according to the following parameters; Qseqid; query (e.g., gene) sequence id, sseqid; subject (e.g., reference genome) sequence id, pident; percentage of identical matches, length; alignment length, mismatch; number of mismatches, gapopen; number of gap openings, qstart; start of alignment in query, qend; end of alignment in query, sstart; start of alignment in subject, send; end of alignment in subject, evalue; expect value, bitscore; bit score.

Supplementary Table 3. Evaluation of *trn*H DNA barcoding region.

qseqid	sseqid	pident	length	mismatch	gapopen	qstart	qend	sstart	send	evalue	bitscore
Nubaria1	YP_009141593.1	100	288	0	0	1	864	144	431	0	559
Magna901	YP_009662972.1	100	278	0	0	1	834	154	431	0	570
Super_Supreme	YP_009662972.1	100	266	0	0	1	798	154	419	0	548
Sirinafa	CED95595.1	80.46	87	17	0	2	262	153	239	2.34E-53	145
Cuf101	ALH22171.1	82.52	143	21	2	27	455	25	163	3.34E-71	223
Perfect	QBJ26779.1	87.62	105	13	0	88	402	36	140	9.01E-56	187
SW9623	BAO66622.1	100	229	0	0	1	687	39	267	1.63E-165	467
Ismailia1	CCI55129.1	100	288	0	0	1	864	147	434	0	587

Sequence estimates of DNA barcoding locus of *trn*H gene were analyzed according to the following parameters; Qseqid; query (e.g., gene) sequence id, sseqid; subject (e.g., reference genome) sequence id, pident; percentage of identical matches, length; alignment length, mismatch; number of mismatches, gapopen; number of gap openings, qstart; start of alignment in query, qend; end of alignment in query, sstart; start of alignment in subject, send; end of alignment in subject, evalue; expect value, bitscore; bit score.
